# Dirac Nodal Arc Semimetal PtSn_4_: An Ideal Platform for Understanding Surface Properties and Catalysis for Hydrogen Evolution

**DOI:** 10.1002/anie.201906109

**Published:** 2019-08-13

**Authors:** Guowei Li, Chenguang Fu, Wujun Shi, Lin Jiao, Jiquan Wu, Qun Yang, Rana Saha, Machteld E. Kamminga, Abhay K. Srivastava, Enke Liu, Aliza N. Yazdani, Nitesh Kumar, Jian Zhang, Graeme R. Blake, Xianjie Liu, Mats Fahlman, Steffen Wirth, Gudrun Auffermann, Johannes Gooth, Stuart Parkin, Vidya Madhavan, Xinliang Feng, Yan Sun, Claudia Felser

**Affiliations:** ^1^ Max Planck Institute for Chemical Physics of Solids 01187 Dresden Germany; ^2^ School of Physical Science and Technology ShanghaiTech University 201203 Shanghai China; ^3^ Department of Physics, Chemistry and Biology (IFM) Linköping University 58183 Linköping Sweden; ^4^ Max Planck Institute for Microstructure Physics 06120 Halle Germany; ^5^ Zernike Institute for Advanced Materials University of Groningen 9747 AG Groningen The Netherlands; ^6^ Department of Chemistry Carleton College MN 55057 Northfield USA; ^7^ Department of Chemistry and Food Chemistry Technische Universität Dresden 01062 Dresden Germany; ^8^ Department of Physics and Frederick Seitz Materials Research Laboratory University of Illinois Urbana-Champaign Urbana Illinois 61801 USA

**Keywords:** Dirac semimetal, electrocatalysis, hydrogen evolution reaction, PtSn_4_, surface states

## Abstract

Conductivity, carrier mobility, and a suitable Gibbs free energy are important criteria that determine the performance of catalysts for a hydrogen evolution reaction (HER). However, it is a challenge to combine these factors into a single compound. Herein, we discover a superior electrocatalyst for a HER in the recently identified Dirac nodal arc semimetal PtSn_4_. The determined turnover frequency (TOF) for each active site of PtSn_4_ is 1.54 H_2_ s^−1^ at 100 mV. This sets a benchmark for HER catalysis on Pt‐based noble metals and earth‐abundant metal catalysts. We make use of the robust surface states of PtSn_4_ as their electrons can be transferred to the adsorbed hydrogen atoms in the catalytic process more efficiently. In addition, PtSn_4_ displays excellent chemical and electrochemical stabilities after long‐term exposure in air and long‐time HER stability tests.

## Introduction

The hydrogen evolution reaction (2 H^+^ + 2 e^−^ → H_2_, HER) that occurs at the solid–liquid interface plays a vital role in clean energy conversion and for the understanding of other complicated heterogeneous reactions. The use of suitable catalysts that have high efficiency, selectivity, and stability is crucial for practical applications.^1^ Many advanced electrocatalysts have been designed and developed by employing a variety of strategies.^2^ However, a suitable lower cost replacement of the benchmark Pt catalyst has not yet been developed. The dominance of Pt as the best catalyst is an inevitable result of several key characteristics. 1) The excellent chemical stability of Pt provides active sites for obtaining a high turnover frequency (TOF). 2) Its ultra‐high electrical conductivity guarantees fast electron transport and reduces the Schottky barrier at the interface between the Pt and adsorbates. 3) A suitable d‐band center favors the metal‐adsorbate interaction and hydrogen release process.^3^

Recent observations of unoccupied metallic topological surface states (TSSs) suggest that certain noble metals, such as Pt are in fact *Z*_2_ topological metals.^4^ The metallic surface states of these metals are protected by time reversal symmetry and are robust against surface modification and defects under ambient conditions.^5^ These are precisely the conditions that are beneficial for the various surface reactions that occur on the crystal surface.^6^ Our previous study on a range of topological materials from bismuth chalcogenide topological insulators^7^ to Weyl semimetals,^8^ demonstrated that each of these could be promising candidates for use as catalysts.

However, both topological insulators and Dirac/Weyl semimetals exhibit very low carrier densities around the Fermi level owing to weak electrostatic screening. We approach this problem by considering a topological nodal arc. In the case of Dirac node arc semimetals, the band crossing points around the Fermi level form a Dirac node arc in momentum space. Many exotic properties, such as ultrahigh magnetoresistivity, carrier mobility are related to the Dirac fermions and topological surface states.^9^ This suggests the following question: can the Pt atomic layer be combined with the exotic physical properties related to the Dirac node arc to speed up the HER process?

To pursue this question, we examined the bulk single crystal Dirac semimetal PtSn_4_. Recently, a high density of conduction electrons and Dirac node arcs were observed in single‐crystalline PtSn_4_ and subsequently confirmed using de Haas‐van Alphen quantum oscillations.9c, 10 The extremely weak bonding between the Sn‐Pt‐Sn layers of the compound enables the exposure of the Pt skin without the presence of metastable dangling bond surface states. We observe an ultra‐low resistivity of only 42 μΩ cm and a high mobility of 200 cm^2^ V^−1^ s^−1^ at room temperature in bulk single‐crystalline PtSn_4_, where the robust surface states are mainly derived from the p orbitals of Pt skin atoms. These lend a considerable contribution to the local density of states (DOS) near the Fermi Level and could act as electron donors for adsorbed molecules. With the bulk single crystal acting as both the cathode and catalyst for the HER, Pt‐like activity is exhibited together with a decrease in Pt usage of 71 % in mass. The TOF value for the PtSn_4_ bulk single crystals reached 1.54 H_2_ s^−1^ at 100 mV, surpassing those of state‐of‐the‐art Pt/C, MoS_2_, and other noble‐metal based catalysts. Of greater importance is the superior electrocatalytic stability of the single crystal after a long‐time and high current density test. These observations may be used to develop a new design principle for high intrinsic electrochemical catalysts with defined active sites.

## Results and Discussion

**Figure 1:**
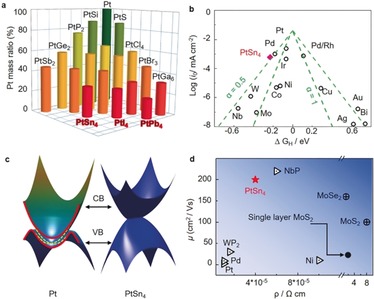
Motivations for searching for advanced electrocatalysts beyond pure Pt: a) The Pt mass ratios in binary compounds that include Pt. b) HER volcano plot for PtSn_4_ and selected metal catalysts. The Gibbs free energy of PtSn_4_ is very close to that of pure Pt. c) Band structure of Pt. The metallic topological surface states are shown in yellow. The bulk states (upper and lower parts) and surface states (middle part) are separated by the red line. The band structure of PtSn_4_ with non‐closed Dirac node arc is also shown. d) The room temperature mobility and conductivity of PtSn_4_ with the selected advanced electrocatalysts.

Starting with Pt, we checked all of the Pt_*x*_ *M_y_* (*M* is a main group element) binary compounds, and selectively short‐listed specific representative cases that has the lowest Pt content with a certain *M* element. As shown in Figure 1 a, PtSn_4_ is chosen for this study because of its facile large‐scale synthesis and high electrical conductivity. As a widely accepted descriptor in prediction HER activity, the Gibbs free‐energy of the intermediate state, |Δ*G*_H*_|, was calculated for different models (Figure S1 in the Supporting Information).^11^ A Pt55 metal cluster was also included for comparison.^12^ As shown in the Volcano plot (Figure 1 b), Pt exhibits the optimal level for H adsorption with a near zero value of Δ*G*_H*_=−0.11 eV, suggesting the best electrocatalytic activity from a thermodynamic viewpoint. In stark contrast, the exposure of the Pt layer in the PtSn_4_ (010) plane resulted in a Δ*G*_H*_ value of −0.28 eV, which is better than that of the Pt55 clusters, with a value of −0.33 eV. These results indicate that the Pt atomic layer of the PtSn_4_ single crystal could be an ideal surface for HERs.

Figure 1 c shows the band structure of Pt, and the topological surface states above the Fermi energy on the (111) surfaces owing to the bulk *Z*_2_ topological order can be observed. Although the *Z*_2_ topological order does not exist in PtSn_4_, it hosts a Dirac nodal arc in the bulk band structure (Figure 1 c).9c This results in a large density of states around the Fermi level. Dirac electrons exist in the sheet, which generally correspond to a large carrier mobility. Figure 1 d shows a comparison of the conductivity and mobility of a selection of advanced HER catalysts, which indicates the advantages of the PtSn_4_ single crystal. The mobilities of the electrons and holes are very similar between 5 and 300 K and reach 200 cm^2^ V^−1^ s^−1^ at room temperature, which is even higher than that of back‐gated MoS_2_, MoSe_2_, WS_2_, WSe_2_, and black phosphorus on SiO_2_ substrates.^13^

**Figure 2:**
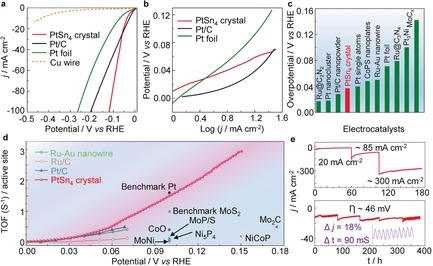
Electrochemical performance of PtSn_4_ single‐crystal catalyst. a) HER polarization curves for the Cu wire, Pt foil, 20 % Pt/C, and PtSn_4_ single crystal. b) Corresponding Tafel plots for the Pt foil, 20 % Pt/C, and PtSn_4_ single crystal in 1 m KOH. c) Overpotential of the PtSn_4_ single‐crystal catalyst at 10 mA cm^−2^ and some recently reported results for HER electrocatalysts. d) TOF values for the PtSn_4_ single‐crystal catalyst and other recently reported results for HER electrocatalysts. e) Current–time (*i*–*t*) chronoamperometric response of the PtSn_4_ electrocatalyst for increasing current densities from 20–300 mA cm^−2^, at an overpotential of 46 mV. The inset shows the changes in the current density during stirring of the electrolyte with a magnetic bar.

With the above theoretical investigation and experimental facts in mind, high quality PtSn_4_ single crystals were grown out of an Sn‐rich binary melt. The HER performance evaluation of this single crystal was carried out in a conventional three‐electrode electrochemical cell in 1 m KOH electrolyte (the details are provided in the Methods section, Figure S2). The obtained polarization curves are shown in Figure 2 a. A sharp increase in the cathode current with an onset overpotential of approximately 0 mV was observed on the PtSn_4_ single crystal, with a burst of gas bubbles that appeared on the crystal surface (Figure S3 and Movie S1). To achieve a current density of 10 mA cm^−2^, a low overpotential of 37 mV is required, which is close to that of the Pt/C nanostructure (28 mV) and much less than that of Pt foil (71 mV). It should be noted that the PtSn_4_ single crystal demonstrates a higher activity than that of even pure Pt foils and Pt/C nanostructures (at higher current densities) in alkaline media, although Pt has a much smaller Δ*G*_H*_ value. This may be caused by the sluggish water dissociation kinetics of Pt in alkaline media, which need not be considered under acidic conditions. Thus, we calculated the water dissociation energy barrier for the Pt (111) and PtSn_4_ (010) surfaces, respectively. Indeed, as illustrated in Figure 4 e, PtSn_4_ has a much lower energy barrier of 0.41 eV than that for Pt (0.91 eV).

A Tafel analysis yields Tafel slopes of 39, 34, and 74 mV dec^−1^. for the PtSn_4_ single crystal, 20 % Pt/C, and Pt foil, respectively, implying a rapid HER rate with a Volmer‐Heyrovsky pathway as the rate‐determining step (Figure 2 b). The calculated HER exchange current density (*j*_0_) for the PtSn_4_ single crystal is 0.585 mA cm^−2^, which is greater than that of the nanostructure Pt/C catalyst (0.518 mA cm^−2^). This indicates that PtSn_4_ is a superior electrocatalyst in alkaline media, with a better performance compared with those of nanostructures such as Pt_3_Ni nanowires (51 mV),^14^ Pt‐Ni excavated nano‐multipods (71 mV),^15^ and PtSe_2_ nanofilms (327 mV)^16^ (Figure 2 c and Table S5).^17^ TOF for each active site of PtSn_4_ is calculated to determine the intrinsic activity (Figure S4, see the Supporting Information). The TOF values of PtSn_4_ at 50 and 100 mV are 0.375 and 1.54 H_2_ s^−1^, respectively, which are greater than those of Pt/C (0.27 H_2_ s^−1^ @ 50 mV) and pure Au‐Ru nanowires (0.31 H_2_ s^−1^ @ 50 mV),^18^ and much greater than those of other nanostructured electrocatalysts (Figure 2 d).^19^ The multiple‐step chrono potentiometric process employed for the PtSn_4_ electrode is displayed in Figure S5, where the current density is increased from 10 to 110 mA cm^−2^, without an *i*R correction. Simultaneous changes in the current densities and applied voltage demonstrated excellent mass transport, conductivity, and mechanical robustness of the single crystalline electrode.^20^

We further investigated the long‐term electrochemical and chemical stability of the single‐crystal electrode. The PtSn_4_ bulk single crystal shows excellent stability for an increasing current of 20 to 300 mA cm^−2^, with a total measurement time of 175 h. At a constant overpotential of 46 mV, the current density is maintained for approximately 400 h without significant activity loss (Figure 2 e). Moreover, the HER activity can be retained even after long‐time air exposure. As shown in Figure S6, the polarization curves were recorded after three‐months of air exposure, and 10 000 cyclic voltammetry sweeps, showing a similar performance to that of the initial tests.

**Figure 3:**
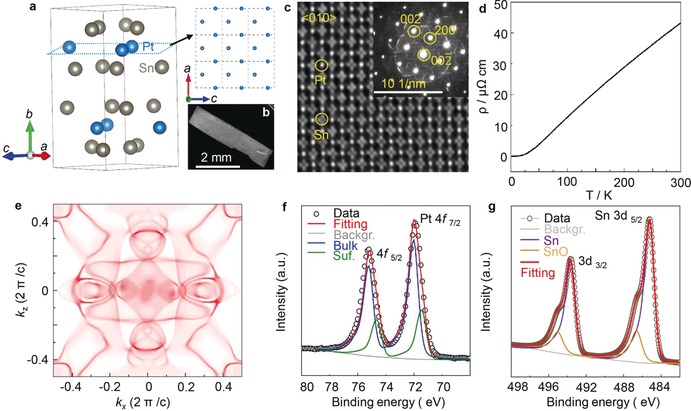
Structure information of bulk single‐crystal PtSn_4_. a) Crystal structure of the PtSn_4_ derived from the single‐crystal XRD at 300 K. The exposed Pt atomic layer was constructed as shown by the upper left panel. b) A typical SEM image of the obtained PtSn_4_ single crystal. c) HRTEM image of the PtSn_4_ sample prepared using the focused ion beam technique (FIB). Inset: the selected area diffraction (SAED) pattern recorded along the [010] crystal orientation. d) Temperature‐dependent resistivity along the [010] direction. An ultralow *ρ* of 42 μΩ cm at room temperature was observed. e) Calculated surface Fermi surface based on the semi‐infinite model. The XPS results for the f) Pt 4f and g) Sn 3d.

To better understand the origin of the HER activity, the structure of the single crystal was determined by single‐crystal X‐ray diffraction (Table S1–S3). The structure was solved in the centrosymmetric space group *Ccca* (No. 68), with lattice parameters *a*=6.4208(7), *b*=11.3592(1), and *c*=6.3766(7) Å at 300 K. Figures 3 a and S7 demonstrate the layered structure of PtSn_4_, which is composed of stacked Sn‐Pt‐Sn layers along the *b* axis with a Pt–Sn distance of 2.7905(9) Å (selected crystallographic data are provided in the Supporting Information, CCDC 1841795 contain the supplementary crystallographic data for this paper. These data can be obtained free of charge from The Cambridge Crystallographic Data Centre.). A homogeneous composition and orientation can be observed from the uniform contrast of the backscatter electron images (Figure 3 b and Figure S8). A thin lamella sample for high‐resolution transmission electron microscopy (TEM) was fabricated by focused ion beam (FIB). Clear lattice fringes with an interplane distance of 0.32 nm, which correspond to the (200) plane of PtSn_4_ can be seen from the lattice‐resolved TEM image (Figure S9a,b). The high quality of the single crystal was further demonstrated by an excellent match between the experimental and simulated electron‐diffraction patterns along the [010] direction (see the inset in Figure 3 c and Figure S10). The atomic structure of the PtSn_4_ single crystal was further investigated with noise corrected high‐resolution scanning TEM (HR‐STEM) (Figure 3 c). The Sn‐Pt‐Sn stacking sequence was retained, with a 100 % occupancy of the Pt and Sn sites.

The in‐plane resistivity (*ρ*) of the PtSn_4_ crystal was measured in the temperature range 2–300 K, which displays a linear temperature dependence above 50 K (Figure 3 d), reflecting the dominance of the acoustic phonon scattering over the electron transport. The resistivity exhibits an ultralow value of *ρ*≈42 μΩ cm at room temperature, which is as low as the values of pure Pt and Sn. A very large residual resistivity ratio *RRR*=*ρ* (300 K)/ *ρ* (2 K)=1000 was obtained, together with a non‐saturating quadratic magneto‐resistivity up to 9 T (Figure S11a), suggesting the high quality of the single crystal. The positive slope of the Hall resistivity as a function of applied magnetic field, as well as the positive thermopower (Figure S11b), indicate that the holes were the dominant charge carriers. This ultrahigh conductivity and mobility may be derived from the unique Fermi surface and band dispersion of the PtSn_4_ single‐crystal, which is a general characteristic of nodal arc semimetals. Indeed, our band structure calculation showed a band crossing just below the Fermi level (Figure S12). The (010) face Fermi surfaces were constructed as shown in Figure 3 e, which consist of large electron and hole pockets.9c

The electronic state of the elements on the PtSn_4_ single‐crystal surface was determined by X‐ray photoelectron spectroscopy (XPS). The XPS survey spectrum confirmed the presence of Pt and Sn (Figure S13). As shown in Figure 3 f, the main peak at a binding energy of 72.0 eV can be assigned to zero‐valence platinum. It is interesting to note that no Pt‐O oxidation states were observed (72.8 eV for Pt^2+^ and 74 eV for Pt^4+^).^21^ The peak at lower binding energy originates from the surface Pt in PtSn_4_ single crystal. This confirms the ultra‐high stability of the Pt sites in the crystal. The main peak with a binding energy of 485.2 eV can be attributed to Sn^0^ 3d_5/2_.^22^ All of these results are consistent with the previous studies on Pt single crystal surface and Pt alloys, where it was claimed that the formation of Pt−O bonding is very difficult.^23^

**Figure 4:**
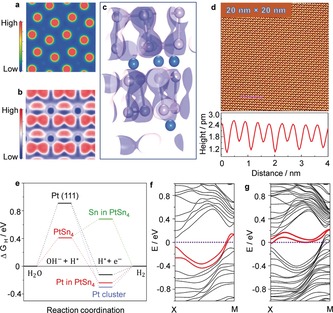
Bonding information in the crystal and the electron transfer mechanism. a) Contour plots of the total charge distribution of PtSn_4_ in the (010) plane. The electronic charges are almost all distributed in the vicinity of the Pt and Sn atoms. b) The 3D‐electron localization function mapping of the (010) surface shows that there is a weak bonding between the in‐plane Sn atoms. c) The 3D‐electron localization function (ELF), with an iso‐surface value of 0.72. d) Atomically resolved STM topography of the PtSn_4_ (010) surface. Inset: the corresponding fast Fourier transform (FFT). The line profile measured along the pink line in (d) indicates a lattice parameter of 0.451 nm; the bias voltage was *V*_b_=100 mV with a tunnel current of *I*_set_=200 pA. e) The Gibbs free energy diagram of the PtSn_4_ (010) surface with an exposed Sn layer, Pt layer, Pt metal (111) face, and Pt nanocluster (55 atoms) calculated at the equilibrium potential of different models, and the water dissociation energy berries on the Pt (111) and PtSn_4_ (010) surfaces. f) Band structure of the PtSn_4_ (010) surface with an exposed Pt layer, but without H adsorption. The robust surface states are along the direction Χ → Μ in the Brillouin zone, located just below the Fermi energy. g) Evolution of the Pt derived surfaces states after hydrogen adsorption when exposing the Pt layer. The surface states donate electrons to the adsorbed H 1s orbital, and shift above the Fermi level.

To understand the bonding character, the electron density distribution and electron localization function (ELF) were calculated. The total charge density distribution map is shown in Figure 4 a,b, demonstrating that the electronic charge is almost completely distributed in the vicinity of the Pt and Sn atoms. This indicates the presence of a metallic bond character in the compound.^24^ The ELF iso‐surface value of 0.72 further highlights the metallic nature of the bonding between Pt and Sn, and suggests a certain amount of electron localization between the intralayer Sn atoms (Figure 4 c). The relatively high ELF value between the Sn atoms indicates the weak covalent nature of the Sn‐Sn bonds. Finally, to confirm the exposure of the Pt layer, scanning tunneling microscopy (STM) was used to examine the in situ surface structure of the PtSn_4_ (010) surface (Figure S14a). The atomically resolved image in Figure 4 d visualizes a square Pt (010) net with an interatomic distance of 0.45 nm at various points in the spirals, which is identical to the bulk orientation and atomic model of the PtSn_4_ (010) face, and in perfect agreement with the value obtained by single‐crystal X‐ray diffraction. This is further demonstrated by the significant difference in the Δ*G*_H*_ values of the Sn and Pt layers (Figure 4 e).

The ultrahigh chemical stability and defined crystal surface of PtSn_4_ provides a good opportunity to investigate the related activity mechanism. DFT calculations were performed on the exposed (010) surface of the PtSn_4_ single crystal. First, the exposure of Sn atoms was considered (the computation details are provided in Supporting Information). Figure S15 displays the calculated surface band structure for the (010) surface with an exposed Sn layer. The abrupt breaking of the weak Sn–Sn bonding creates a Sn‐ derived occupied *s*‐type surface state that typically covers a broad energy range of the surface Brillouin zone. After hydrogen adsorption, the surface states corresponding to the Sn–H occupied bonding states are submerged into the bulk valence bands (Figure S16), with the H s orbital observed in the deep bulk band (Figure S17). However, when the Pt layer is exposed, the surfaces could not be passivated or eliminated by hydrogen as there is no Fermi level pinning at the crystal surface. Indeed, as shown in Figure S18, we observe surface states along the Χ–Μ axis of the surface Brillouin zone (Figures 4 f and Figure S18). The orbital analysis showed that the surface states are mainly formed of p‐like electronic states from the topmost Pt layer (Figures S19a and S19b). To investigate their interaction with hydrogen, the band structures of the hydrogen‐covered surface were also determined. The hydrogen adsorption shifts the surface states above the Fermi level (Figure 4 g and Figure S20). This indicates the transfer of electrons from the surface state to the hydrogen s‐like orbitals. The downshift of hydrogen bands to a position far below the Fermi energy provided further proof of this electron transfer (Figure S21).

## Conclusion

The bulk single‐crystal PtSn_4_ exhibits Pt‐like electrocatalytic activity as a catalyst for HERs. The ultra‐high chemical and electrochemical stability presented by this compound, together with a significant decrease in Pt usage, makes it an ideal candidate to replace Pt catalysts. The linear band crossing in this semimetal provides large room‐temperature carrier mobility and conductivity, which can be attributed to the high Fermi velocity of massless Dirac states.^25^ This facilitates the rapid charger transfer in the catalysis process and thereby increases the HER kinetics. Intrinsic and stable surface states were found in the Pt layer of the PtSn_4_ single crystal. This led to the direct donation of electrons from the occupied robust surface states to adsorbed hydrogen. Rather than adopting the traditional approaches of creating active sites by nanosizing, doping, straining, and edging, the present study indicates that the manipulation of robust surface states, as well as providing high conductivity and mobility, may be used to provide a new guide for obtaining high‐performance catalysts.

## Conflict of interest

The authors declare no conflict of interest.

## Supporting information

As a service to our authors and readers, this journal provides supporting information supplied by the authors. Such materials are peer reviewed and may be re‐organized for online delivery, but are not copy‐edited or typeset. Technical support issues arising from supporting information (other than missing files) should be addressed to the authors.

Supplementary

Supplementary
