# Supplementary material for: Dirac Nodal Arc Semimetal PtSn4: An Ideal Platform for Understanding Surface Properties and Catalysis for Hydrogen Evolution
Source: Angew Chem Int Ed Engl. 2019 Aug 13;58(37):13107–12. doi: 10.1002/anie.201906109 (PMC6772105; doi:10.1002/anie.201906109)
Supplement: Supplementary file 1 — Supplementary [file ANIE-58-13107-s001.pdf]

Angewandte Chemie
Eine Zeitschrift der Gesellschaft Deutscher Chemiker

Supporting Information

Dirac Nodal Arc Semimetal $PtSn_4$: An Ideal Platform for Understanding Surface Properties and Catalysis for Hydrogen Evolution

Guowei Li[+], Chenguang Fu[+], Wujun Shi[+], Lin Jiao, Jiquan Wu, Qun Yang, Rana Saha, Machteld E. Kamminga, Abhay K. Srivastava, Enke Liu, Aliza N. Yazdani, Nitesh Kumar, Jian Zhang, Graeme R. Blake, Xianjie Liu, Mats Fahlman, Steffen Wirth, Gudrun Auffermann, Johannes Gooth, Stuart Parkin, Vidya Madhavan, Xinliang Feng, Yan Sun[+],* and Claudia Felser[+]**

anie_201906109_sm_miscellaneous_information.pdf

Author Contributions

C.F. Investigation: Equal.

Experimental Section

Materials synthesis. $PtSn_4$ single crystal was grown out of a Sn-rich binary melt.[1] High-purity starting elements Pt (ingot, 99.99%) and Sn (ingot, 99.999%) were mixed together with an initial stoichiometry of Pt_4Sn_{96}, and the mixture was subsequently placed in an alumina crucible and sealed in a quartz tube under partial Ar pressure. The quartz tube was heated to 600 °C over a period of 5 h, and kept at this temperature for 20 h. Finally, the tube was slowly cooled down to 350 °C over 60 h.

Characterization. Single crystal X-ray diffraction (XRD) measurements were performed using a Bruker D8 Venture diffractometer equipped with a Triumph monochromator and Photon100 area detector, operating with Mo Kα radiation. Crystal structures at different temperatures are deposited in the Cambridge Structural Database (CSD) of the Cambridge Crystallographic Data Center (CCDC) and can be obtained from www.ccdc.cam.ac.uk (100 K: CCDC 1841794; 100 K: CCDC 1841795). For the transmission electron microscopy (TEM) investigation, we have prepared lamella (6.7 μm × 4.4 μm) from a single crystal of $PtSn_4$ by means of a Ga^+ focused ion beam (FIB) milling instrument [FEI Nova Nanolab 600 SEM/FIB] operating at 0.2 keV-30 keV ion beam energy. Lamella was taken out from the bulk single crystal using the standard lift-out procedure and transferred to a copper grid. Then the lamella was thinned down to the desired thickness. Finally, both the faces of lamella were polished with lower Ga^+ ion-beam energy (5 keV) to reduce the thickness of the damage layer caused by Ga^+ ion implantation and crystal structure amorphization. The longitudinal electrical resistance measurement was conducted using a standard four-probe method with the AC transport option in a PPMS system, with an applied AC current of 16 mA. For all of the transport measurements, the magnetic field was applied along the b-axis, perpendicular to the a–c plane. The XPS spectra were obtained from a UHV surface analysis system equipped with a Scienta-200 hemispherical analyzer. The base pressure of the sample analysis chamber was 2×10^{-10} mbar. To calibrate XPS spectrometer at the right binding energy related to the Fermi level, we use a clean gold film to measure the position of the Fermi level and Au $4f_{7/2}$ peak. The former is at zero binding energy, the latter is at 84.00 eV. Meanwhile, we also check the Ag $3d_{5/2}$ peak at 368.27 eV and Cu $2p_{3/2}$ at 932.67 eV.

Electrocatalytic characterization. The HER catalytic measurements were performed on an Autolab PGSTAT302N with an impedance module electrochemistry workstation. A conventional three electrode cell configuration was employed. An Ag/AgCl (3 M KCl) electrode was used as the reference electrode, and a graphite rod was used as the counter electrode. The bulk $PtSn_4$ single crystal (1 mm × 5 mm × ~ 0.1 mm) was attached to a Cu wire with silver paint and served as working electrode and catalyst. The electrolyte was an Ar saturated 1 M KOH solution. Linear sweep voltammograms were recorded using a $PtSn_4$ single crystal electrode (1 mm × 5 mm) with a scan rate of 1 mV / S. All potentials were referenced to a reverse hydrogen electrode (RHE). The stability tests were performed at an overpotential of 39 mV in the initial test, and at 68 mV after air exposure for three months, corresponding to current densities of 10 and 30 mA cm^{-2}, respectively. A much longer stability test was

carried out with a constant overpotential of 46 mV. The electrolyte is refreshed every three days. The electrolyte is stirred at a speed of ~ 200 rpm (IKA Magnetic Stirrers).

Estimation of electrochemical active surface area (ECSA) and Turnover frequency calculations (TOF)

Since the effective surface area of the catalyst is linearly proportional to the double layer capacitance (C_{dl}), we measured the capacitive currents of the single crystal electrode in the potential range of 0.19 ~ 0.29 V vs. RHE with various scan rates (80, 100, 110, 120, 130, 140 mV/s, etc), where no faradic processes are happened. The specific capacitance is determined by plotting the capacitive currents as a function of scan rate. The specific capacitance can be converted into an electrochemical active surface area (ECSA) using the specific capacitance value for a flat standard with 1 cm^2 of real surface area.

According to the XPS and calculations, we assumed that the Pt is the active center and all the exposed Pt atoms in the (001) plane are possible active sites.

The total number of hydrogens turn overs was calculated from the current density according to:[2]

$$\# H_2 = \left(j \, \frac{mA}{cm^2}\right)\left(\frac{1\, Cs^{-1}}{1000\, mA}\right)\left(\frac{1\, mol\, e^-}{96485.3\, C}\right)\left(\frac{1\, mol\, H_2}{1\, mol\, e^-}\right)\left(\frac{6.022 \times 10^{23}\, H_2}{1\, mol\, H_2}\right) = 3.12\ \times 10^{15}\, \frac{\mathrm{H_2}/s}{cm^2}\, per\, \frac{mA}{cm^2}$$

Then the HER turnover frequency (TOF) as a function of current density is defined as:

$$\mathrm{TOF}\ = \frac{\left(3.12\ \times 10^{15}\, \frac{\mathrm{H_2}/s}{cm^2}\, per\, \frac{mA}{cm^2}\right) \times\ |j|}{\#\, active\ sites\ \ \times A_{ECSA}}$$

Crystal size	0.12 × 0.06 × 0.02 mm^3
Wavelength	0.71073 Å (Mo Kα radiation)
Refinement method	full matrix least squares on F^2, anisotropic displacement parameters
Absorption correction	multi-scan

**Temperature**	**100(2) K**	**300 (2) K**
crystal system	orthorhombic	orthorhombic
space group	*Ccca* (no. 68)	*Ccca* (no. 68)
symmetry	centrosymmetric	centrosymmetric
Z	4	4
D (calculated) (g/cm3)	9.652	9.567
F(000)	556	556
a (Å)	6.4045(7)	6.4208(7)
b (Å)	11.3087(15)	11.3592(16)
c (Å)	6.3646(7)	6.3766(7)
α (°)	90.0	90.0
β (°)	90.0	90.0
γ (°)	90.0	90.0
volume (Å^3)	460.97(9)	465.08(10)
absorption coefficient (mm^{-1})	51.405	50.951
min / max transmission factor	0.1225 / 0.3706	0.1228 / 0.3711
θ range (degrees)	3.60 – 36.97	3.59 – 36.85
index ranges	-8 < h < 8	-8 < h < 8
	-14 < k < 14	-15 < k < 15
	-8 < l < 8	-8 < l < 8
data / restraints / parameters	287 / 0 / 13	294 / 0 / 13
GooF on F^2	1.197	1.199
no. total reflections	4675	4080
no. unique reflections	287	294
no. obs Fo > 4σ(Fo)	240	239
R_1 [Fo > 4σ(Fo)]	0.0289	0.0313
R_1 [all data]	0.0367	0.0420
wR_2 [Fo > 4σ(Fo)]	0.0634	0.0682
wR_2 [all data]	0.0666	0.0733

Table S1. Crystallographic and refinement parameters of $PtSn_4$, measured at 100 K and 300 K.

100 K

Atom	*x*	*y*	*z*	U_{eq} (Å^2)
Pt	0.00000	0.25000	0.25000	0.0068(2)
Sn	0.33436(13)	0.12338(7)	0.08470(13)	0.0073(2)

300 K

Atom	*x*	*y*	*z*	U_{eq} (Å^2)
Pt	0.00000	0.25000	0.25000	0.0099(3)
Sn	0.33403(15)	0.12340(7)	0.08410(15)	0.0124(3)

Table S2. Fractional atomic coordinates of the asymmetric unit and equivalent thermal displacement parameters.

100 K

Interatomic distance (Å)		
Pt	Sn_$4	2.7773(8)
Pt	Sn_$7	2.7773(8)
Pt	Sn_$9	2.7773(8)
Pt	Sn_$1	2.7773(8)
Pt	Sn	2.7826(9)
Pt	Sn_$8	2.7826(9)
Pt	Sn_$5	2.7826(9)
Pt	Sn_$3	2.7826(9)

300 K

Interatomic distance (Å)		
Pt	Sn_$4	2.7825(9)
Pt	Sn_$7	2.7825(9)
Pt	Sn_$9	2.7825(9)
Pt	Sn_$1	2.7825(9)
Pt	Sn	2.7905(9)
Pt	Sn_$8	2.7905(9)
Pt	Sn_$5	2.7905(9)
Pt	Sn_$3	2.7905(9)

Table S3. Selected interatomic distances.

Operators for generating equivalent atoms:

$1 -x+1/2, -y+1/2, -z
$2 -x+1/2, -y, z
$3 -x, -y+1/2, z
$4 x-1/2, y, -z
$5 -x, y, -z+1/2
$6 -x+1, y, -z+1/2
$7 x-1/2, -y+1/2, z+1/2
$8 x, -y+1/2, -z+1/2
$9 -x+1/2, y, z+1/2

Associated content

Supporting Information includes crystallographic information files of $PtSn_4$, measured at 100 K and 300 K (CIF).

Computation details

Density functional theory (DFT) method as implemented in the Vienna ab initio Simulation Package (VASP) was used for the electronic-structure calculations. The exchange–correlation was considered in the revised Perdew-Burke-Ernzerhof (rPBE) parameterized generalized gradient approximation (GGA) and spin–orbital coupling (SOC) was included. After H adsorption, the H atoms are fully relaxed until the force less than 10 meV / Å. The energy cutoff is set as 300 eV. The k-points grid is 6 x 6 x 1.

Adsorption of H atom(s) on (001)-Sn or Pt of $PtSn_4$ and (111)-Pt surfaces

For the H adsorption calculations, the surfaces of $PtSn_4$ (010) and Pt (111) are chosen for the consideration of the dominating planes based on the STM and XPS results. The 2 × 2 (111) Pt and (010) $PtSn_4$ surfaces were constructed by cleaving optimized bulk Pt and $PtSn_4$ in a six- layer-thick slab. The differential adsorption energy of H adsorption is chosen to describe the stability of hydrogen according to the equation[3]:

$$\Delta E_H = E(PtSn_4 + n\ H) - E(PtSn_4 + (n-1)\ H) - 1/2\ E(H_2)$$

Where E ($PtSn_4$ + n H) is the total energy of $PtSn_4$ or Pt with the n hydrogen atoms adsorbed on surface, E ($PtSn_4$ + (n-1) H) is the total energy of $PtSn_4$ or Pt with (n-1) hydrogen atoms adsorbed on surface and E (H_2) is the total energy of hydrogen molecule in gas phase. This gives the binding energy of single H atom adsorption when n is equal to 1. For the determination of active sites, the top-, hollow-, and bridge sites are considered for all the cases.

Water dissociation kinetics calculation

The most energy favorable pattern for water molecule and OH + H pairs adsorbing on slab are obtained by geometric optimization. To search a minimal energy path for water decomposition on the slab, a 2 × 2 × 1 supercell for both Pt (111) and $PtSn_4$ (010) is adopted, and the complete LST/QST method embedded in CASTEP as implemented in Materials Studio is employed for searching the transition states. The convergence criterion of force is set to be 0.01 eV/Å.

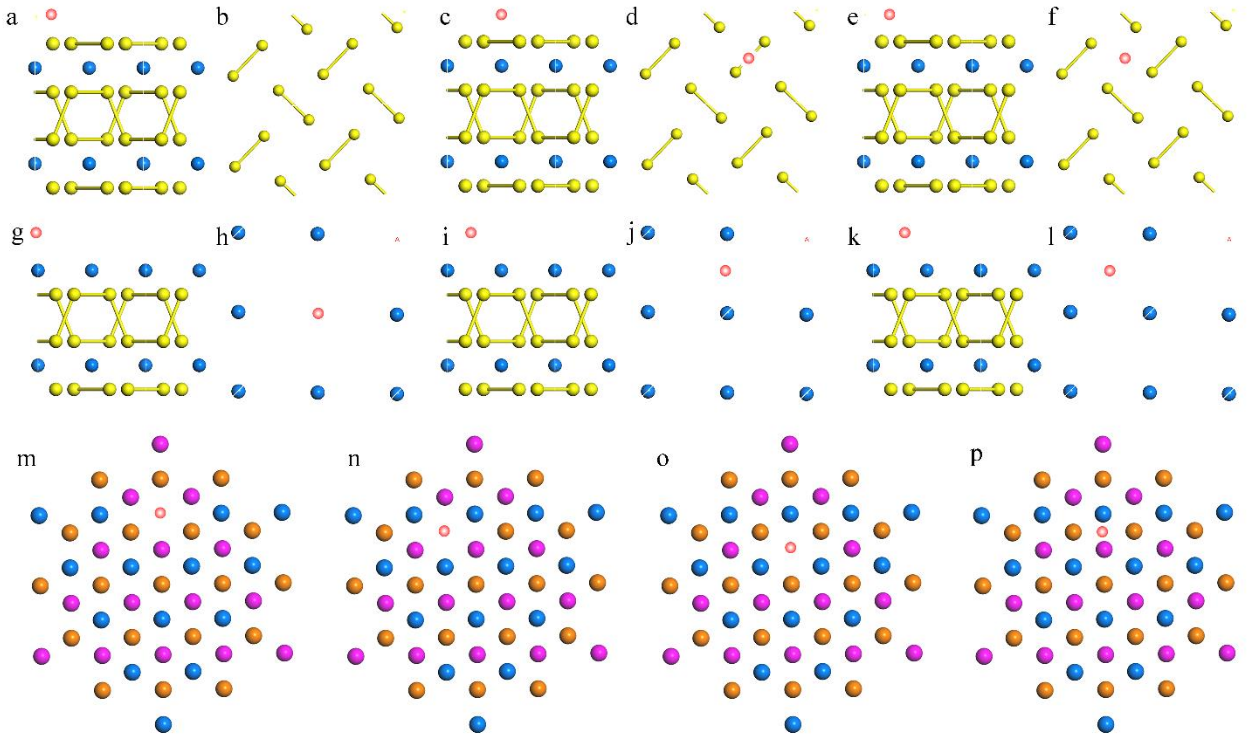

Figure S1. Three possible reaction sites were checked for both (a-f) Sn and (g-l) Pt terminals for $PtSn_4$, both top-view (b, d, f, h, j and l) and side-view (a, c, e, g, I, and k) are given for each site. Blue, yellow and pink atoms represent Pt, Sn, and H atoms, respectively. (m-p) Four possible sites were checked for Pt (111) face, m: top, b: fcc, c; hcp, and p: bridge site. The blue, orange and purple atoms represent first, second and third layer of atoms, respectively.

Catalytic activity of $PtSn_4$ and Pt

The Gibbs free energy ΔG_{H*} of H adsorption on the basal surface of each material are calculated to describe the trends of hydrogen evolution reaction with the following equation:

$$\Delta G_H = \Delta E_H + \Delta E_{ZPE} - T\Delta S_H$$

Here, ΔE_{ZPE} and ΔS_H are the difference in zero points energy and entropy between the adsorbed species and the gas phase molecule, respectively. For the adsorbed species, they can be determined from the vibrational frequencies of the adsorbed species using normal mode analysis with DFT calculation.[4] While for the gas phase molecule, the zero point energy of H_2 and the TS at room temperature (300K) can be looked up from standard molecular tables.[5] A good catalyst for hydrogen evolution is characterized with a ΔG_H value close to 0.

	**H on Sn for $PtSn_4$**	**H on Pt for $PtSn_4$**	**Pt (111)**	**Pt 55**
**ΔG_H**	0.68	-0.28	-0.11	-0.35

Table S4 Gibbs Free energy (eV) of the most stable structure in different materials.

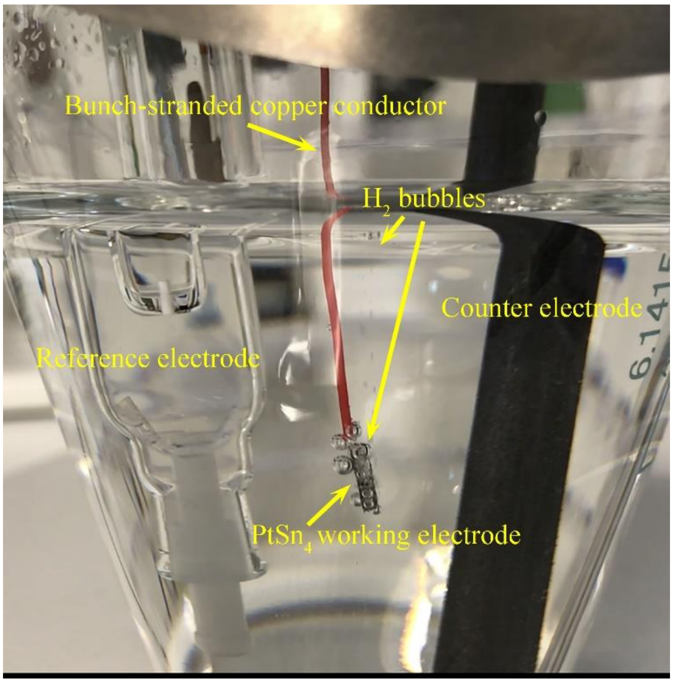

Figure S2. Digital picture of the electrochemical cell setup.

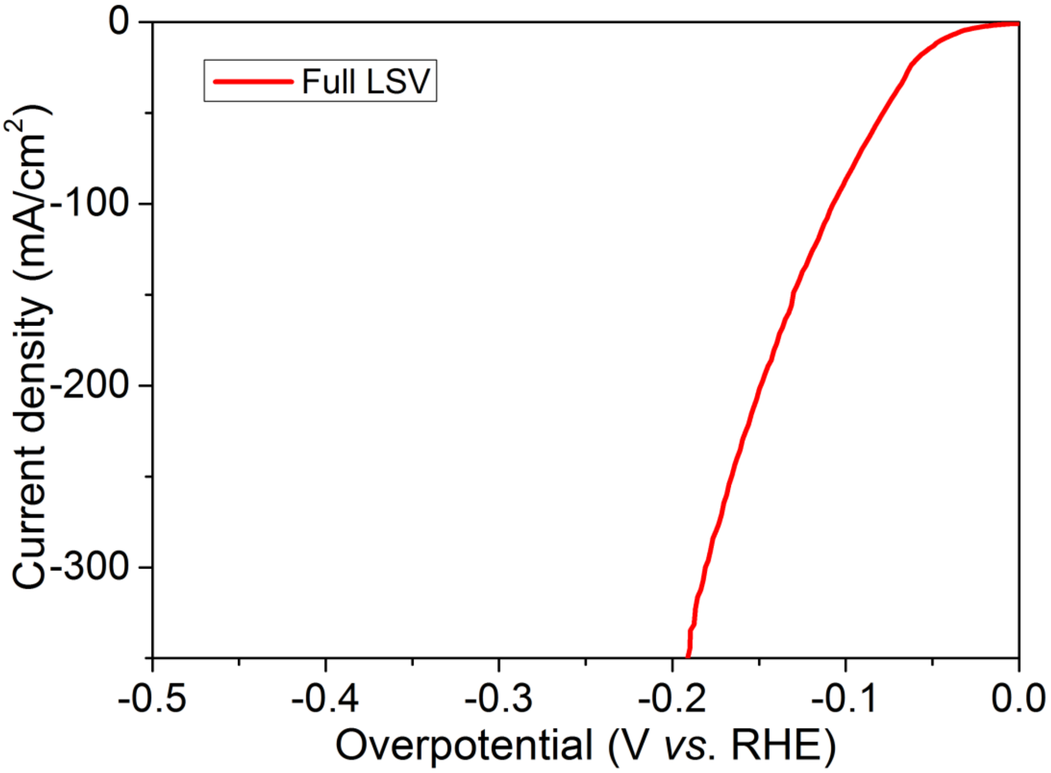

Figure S3. Polarization curves of the $PtSn_4$ single crystal electrode in 1 M KOH solution.

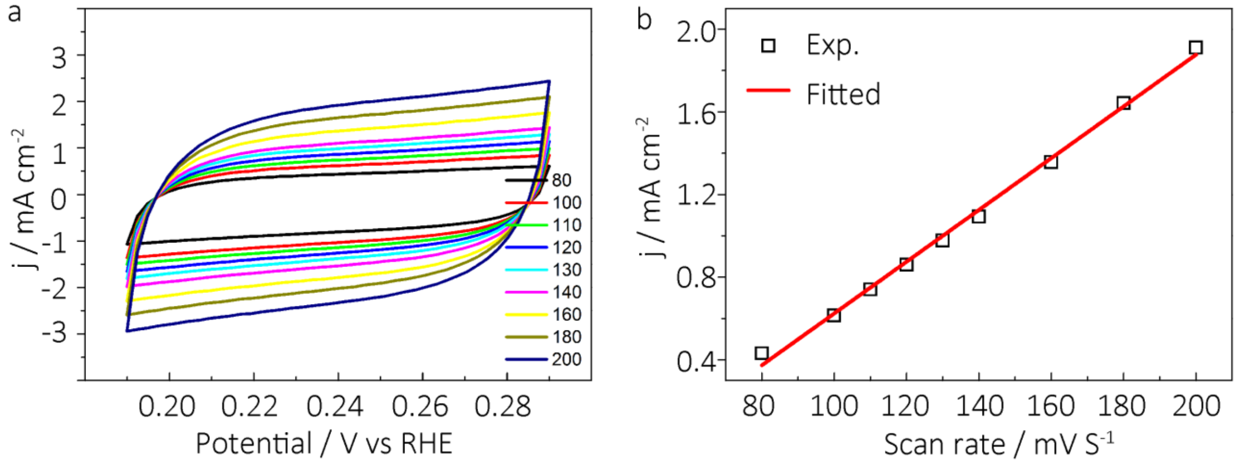

Figure S4. **a.** Electrochemical capacitance measurements to determine the ECSA of $PtSn_4$ catalyst. **b**. The measured capacitive currents plotted as a function of scan rate.

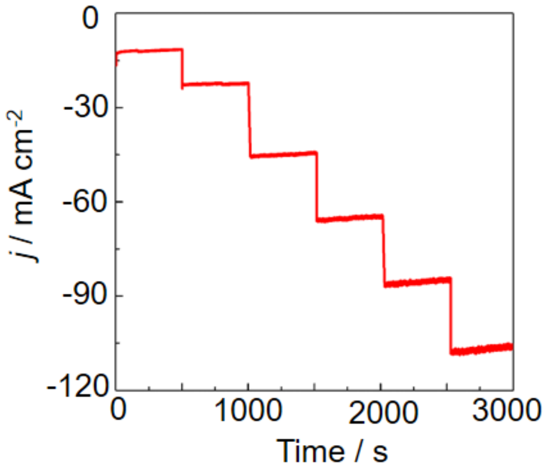

Figure S5. Multi-current process with the current density increased from 10 mA cm^{-2} to 110 mA cm^{-2} without iR correction.

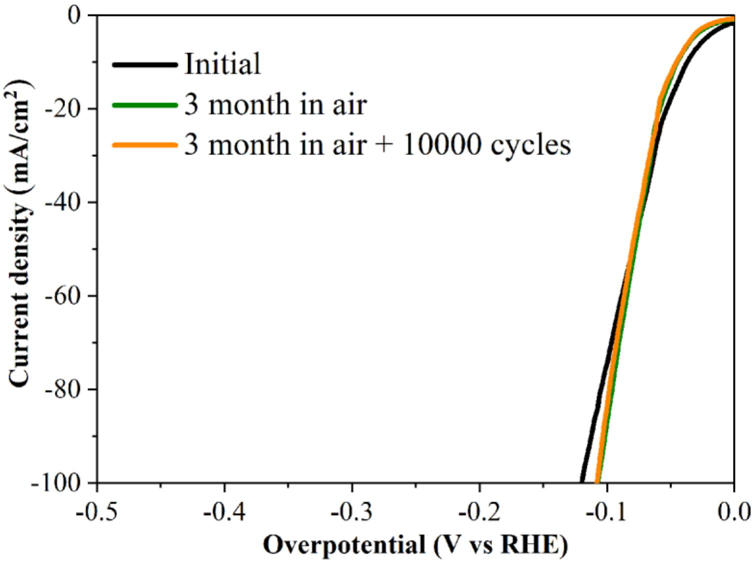

Figure S6. Polarization curves of the $PtSn_4$ single crystal electrode in the initial test, after 3 months, and 10,000 cycles, respectively.

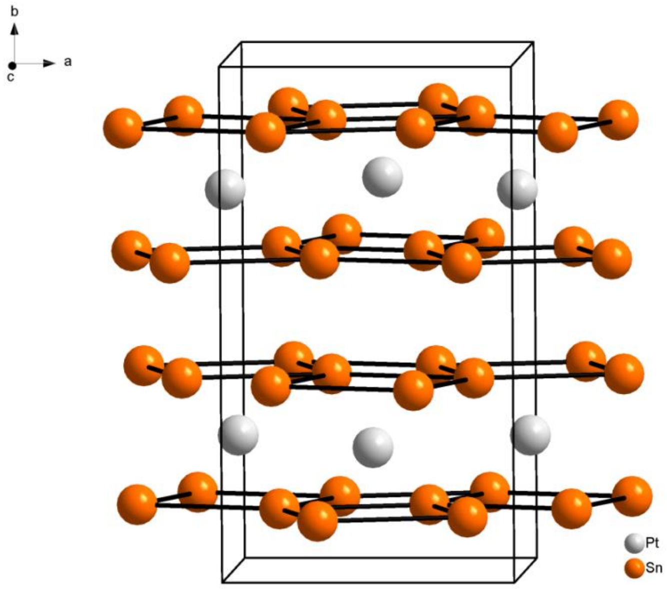

Figure S7. Crystal structure of $PtSn_4$. The chemical bonds are drawn to illustrate the layered nature of the crystal structure, wherein layers of the main group element (Sn) and transition metal (Pt).

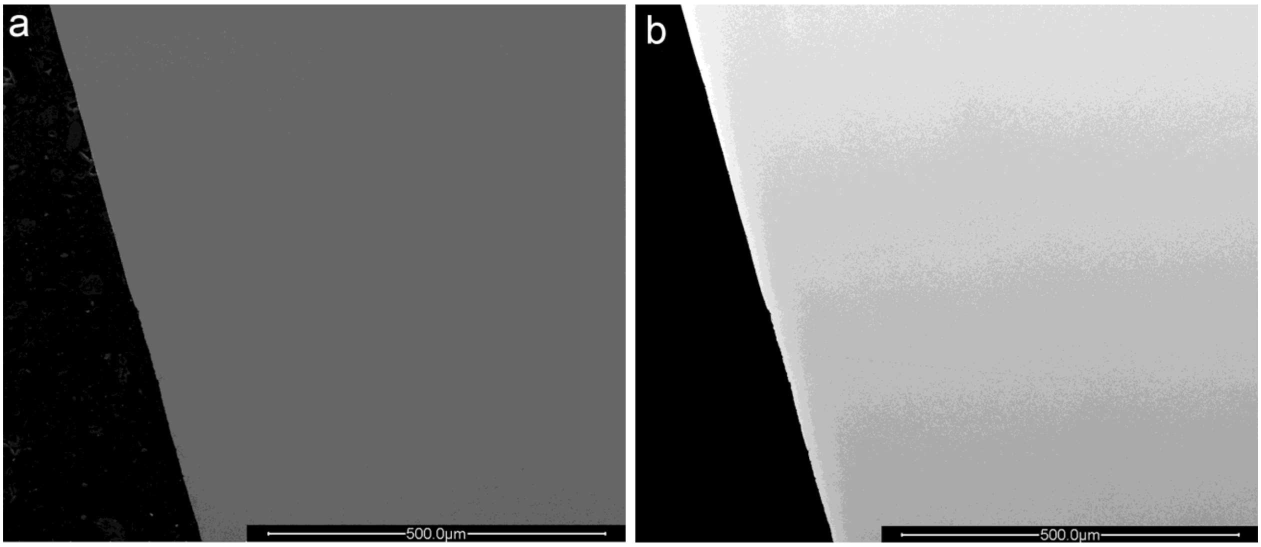

Figure S8. a. Secondary Electron and **b.** Backscatter Electron images of the crystal surface.

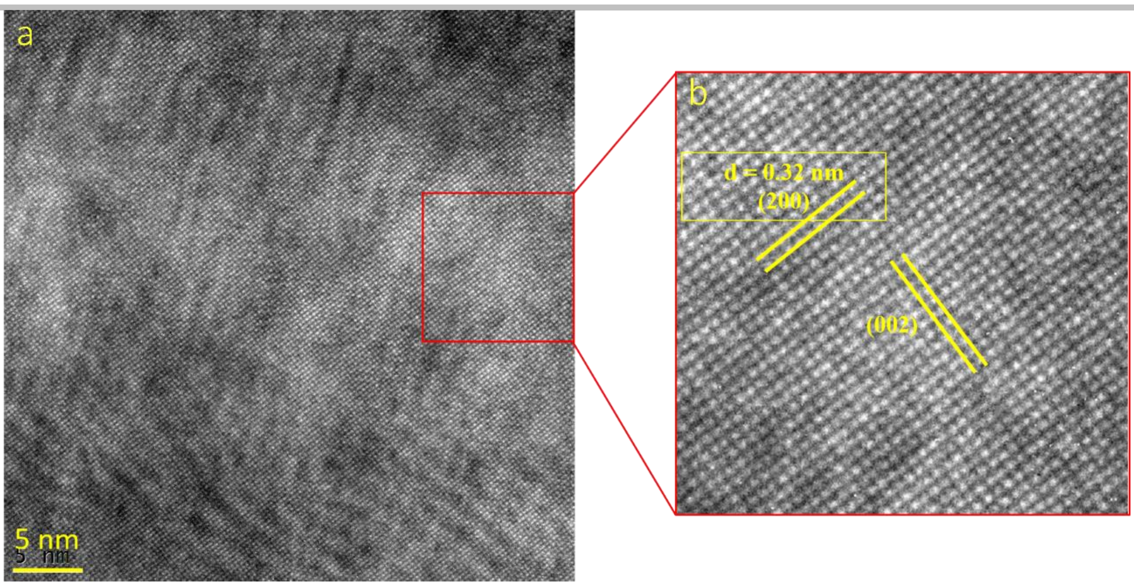

Figure S9. a and b. HRTEM of the $PtSn_4$ single crystal prepared by focused ion beam technique.

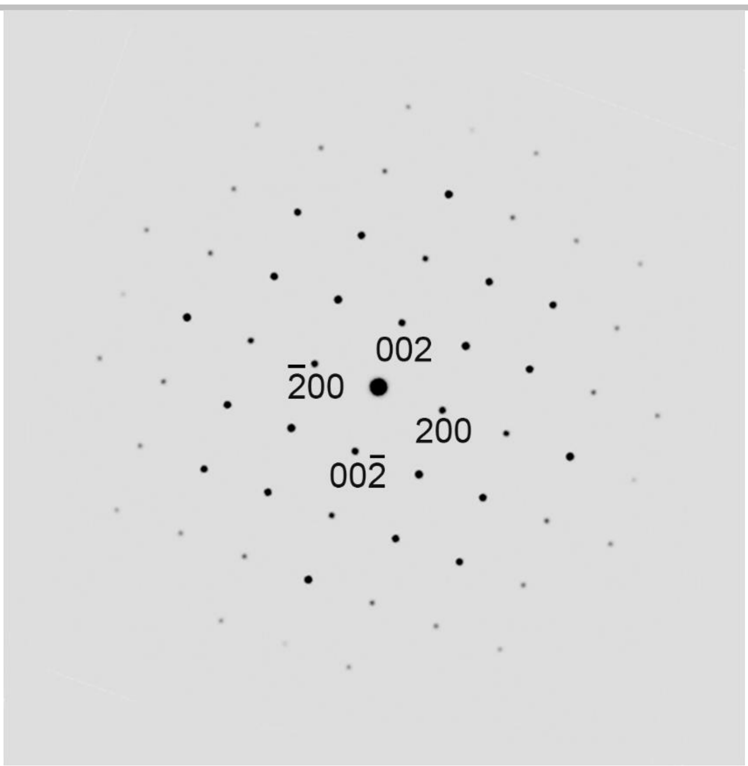

Figure S10. Simulated diffraction pattern along the [010] direction of the $PtSn_4$ single crystal.

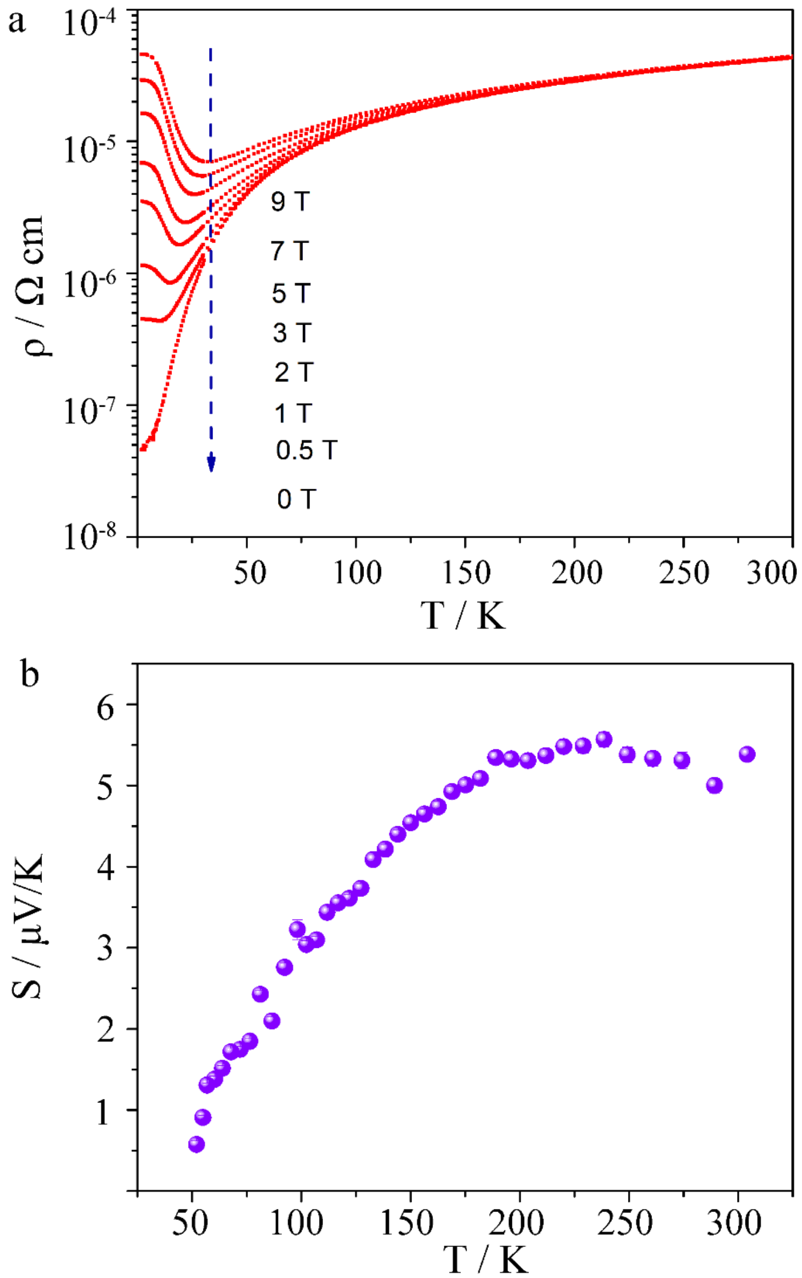

Figure S11. a. Resistivity as a function of temperature T for various magnetic fields B. **b**. The thermopower ***S*** as a function of T.

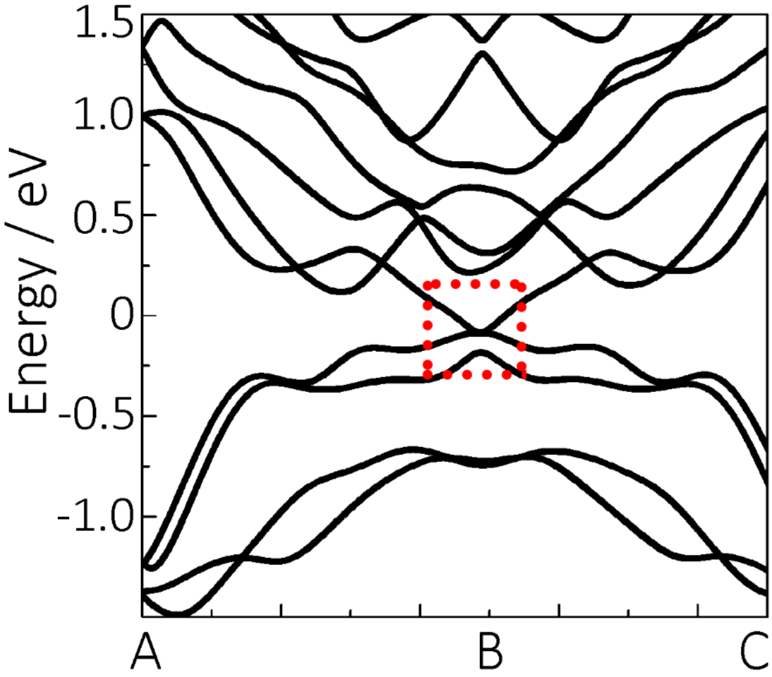

Figure S12. Calculated band structure of the single crystal with band crossing shown in the red box, A (-0.50, 0.10, 0.42), B (-0.50, 0.10, 0.42), C (0.50, 0.10, 0.42) in unit of (2π/a, 2π/b, 2π/c).

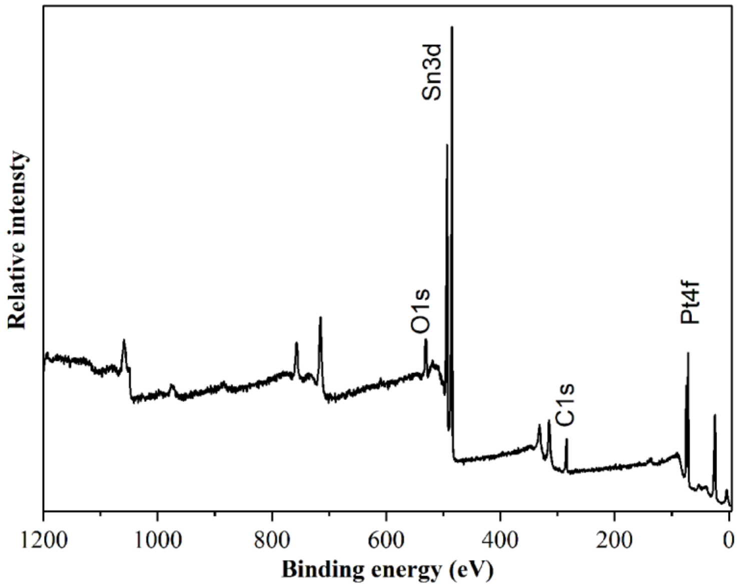

Figure S13. XPS survey spectrum on the bulk $PtSn_4$ single crystal. The appearance of C and O signals indicating the adsorption of small molecular from air on the crystal surface.

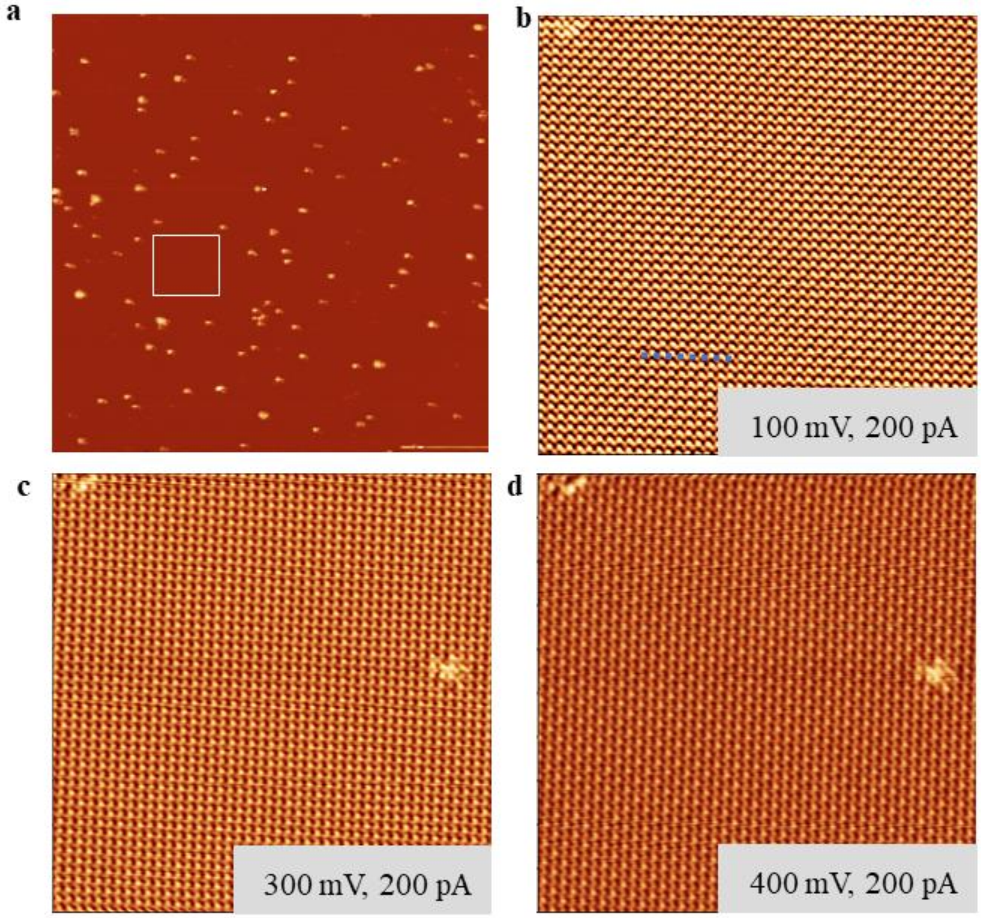

Figure S14. STM image of the cleaved $PtSn_4$ (010) surface, and the corresponding surface with different voltage. **b.** 100 mV, **c.** 300 mV, and **d.** 400 mV.

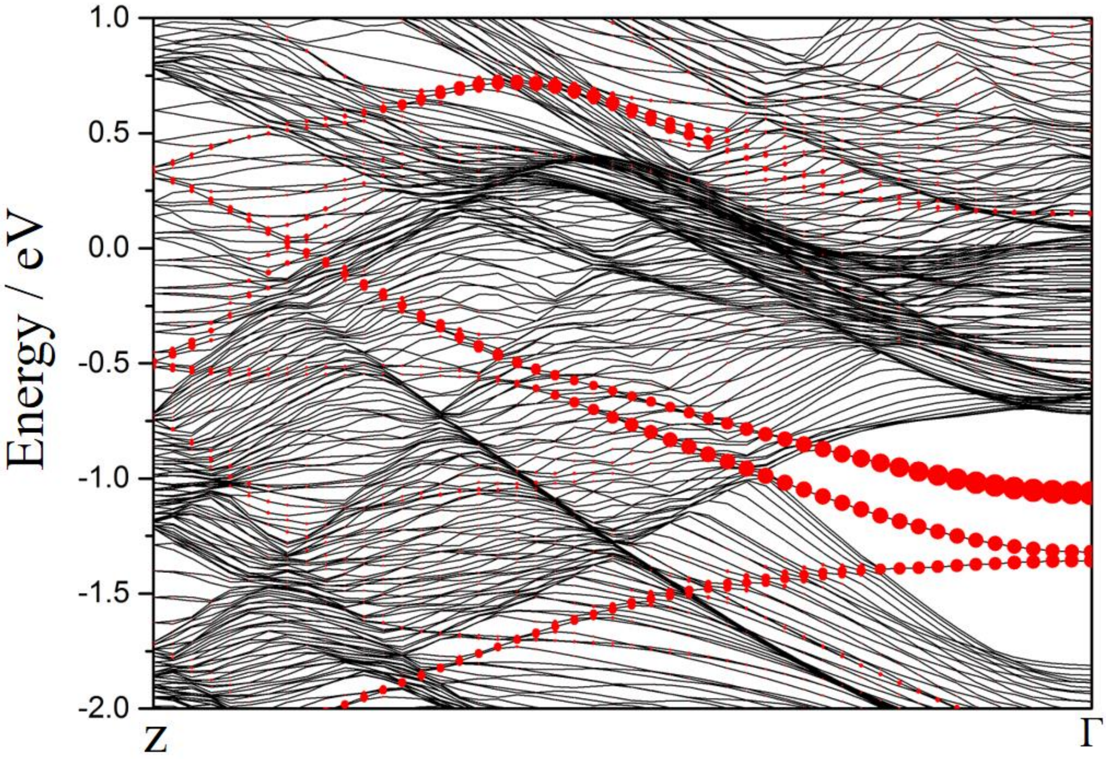

Figure S15. Band structure of the clean $PtSn_4$ (010) surface with Sn terminal

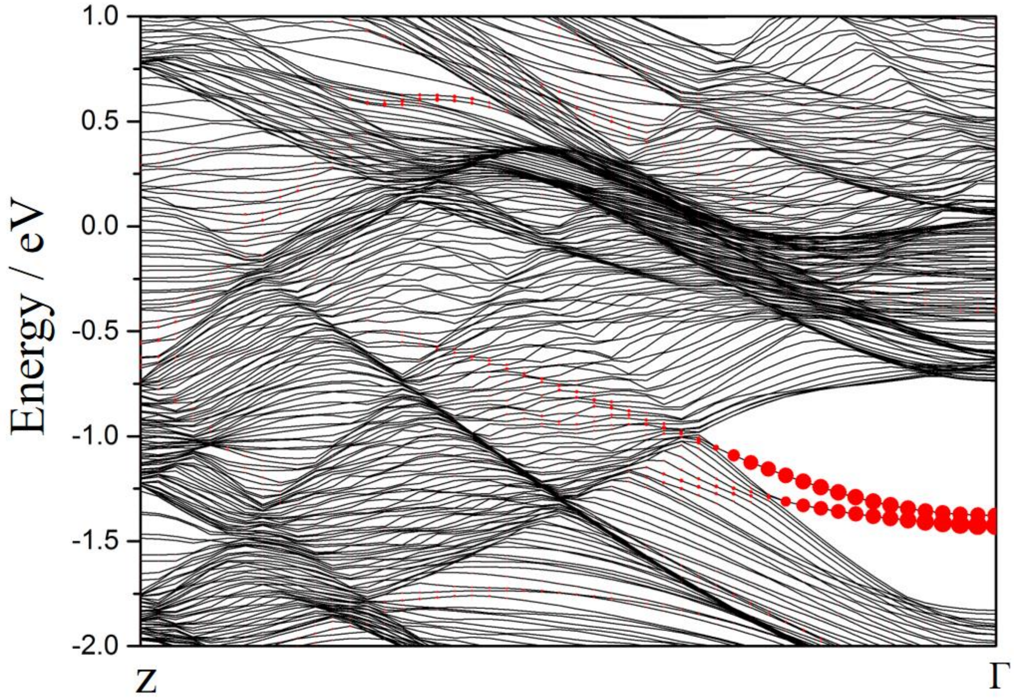

Figure S16. Band structure of the clean $PtSn_4$ (010) surface (red points) with Sn terminal after H adsorption.

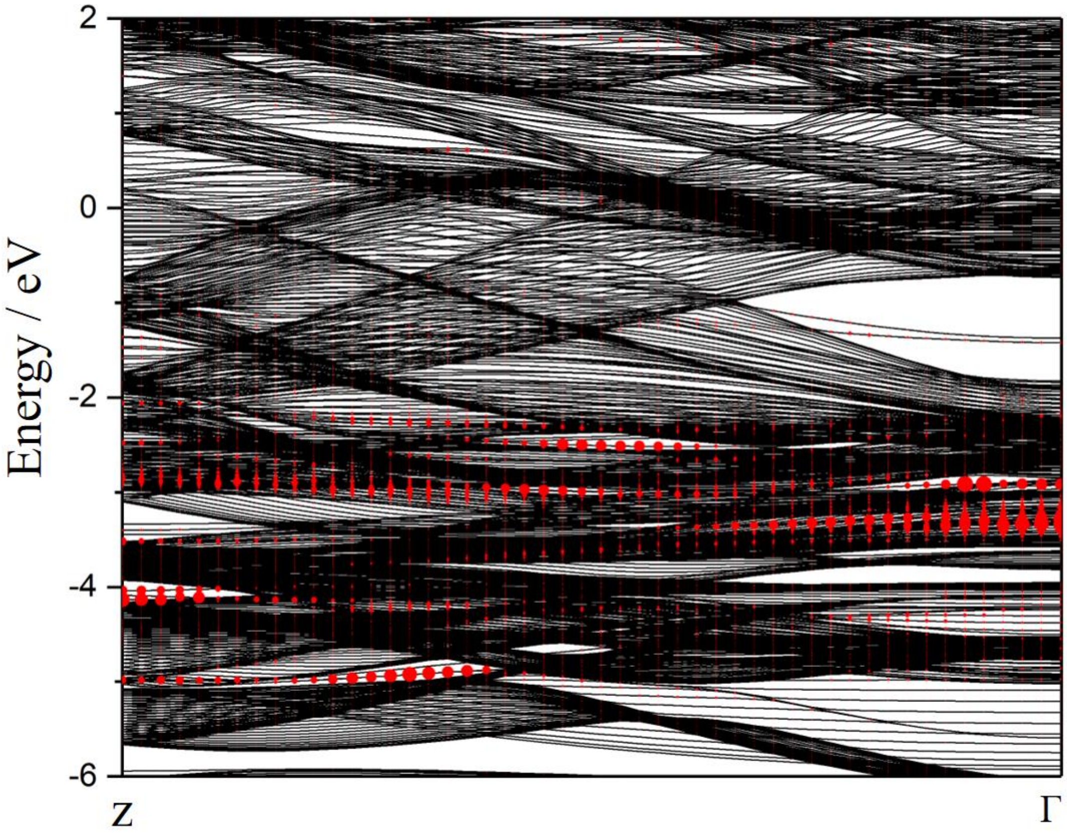

Figure S17. The H orbital after accept electrons from the Sn surface states (red points).

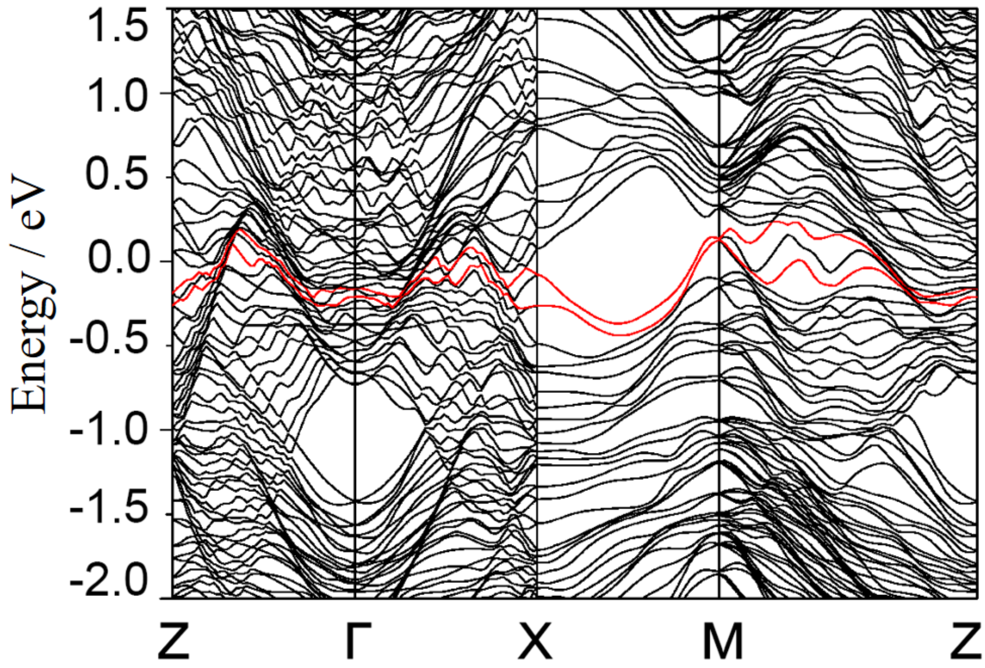

Figure S18. Band structure of the $PtSn_4$ (010) surface without H adsorption. The Pt derived surface states along the direction X→M in the Brillouin zone are shown in red.

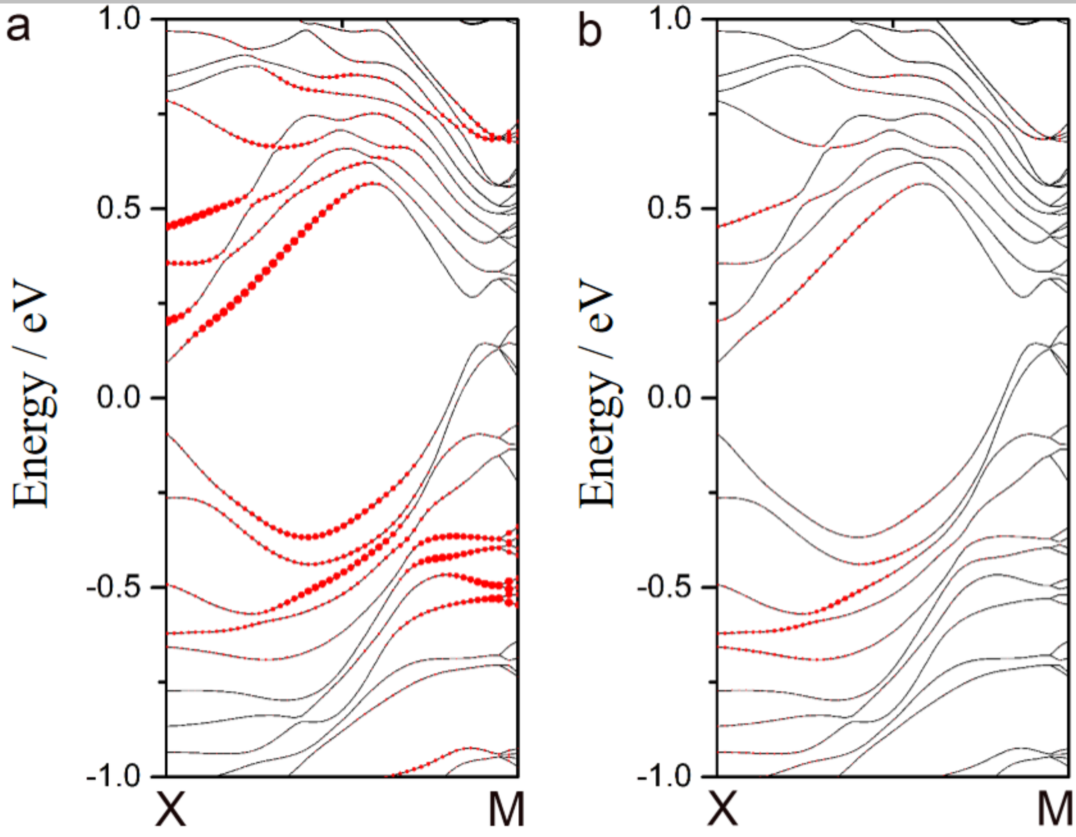

Figure S19. The Pt derived surfaces states are composed of **a.** *p* and **b.** *d* electrons states.

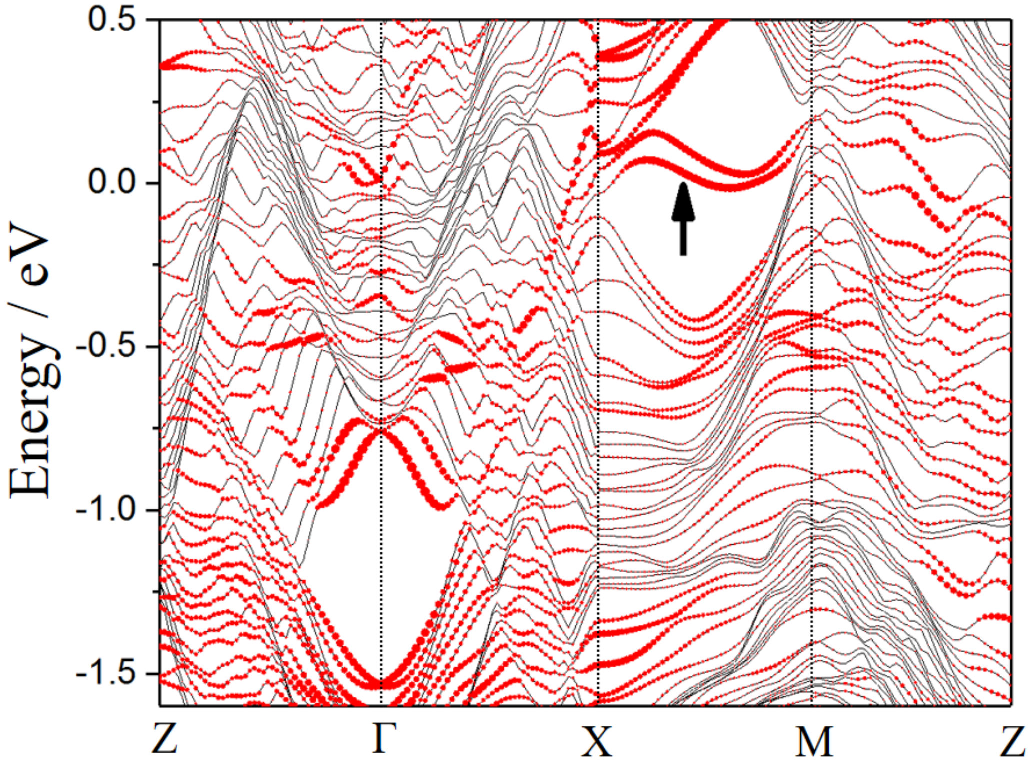

Figure S20. The surfaces states shift to the position higher than the Fermi energy after donate the electrons to adsorbed hydrogen.

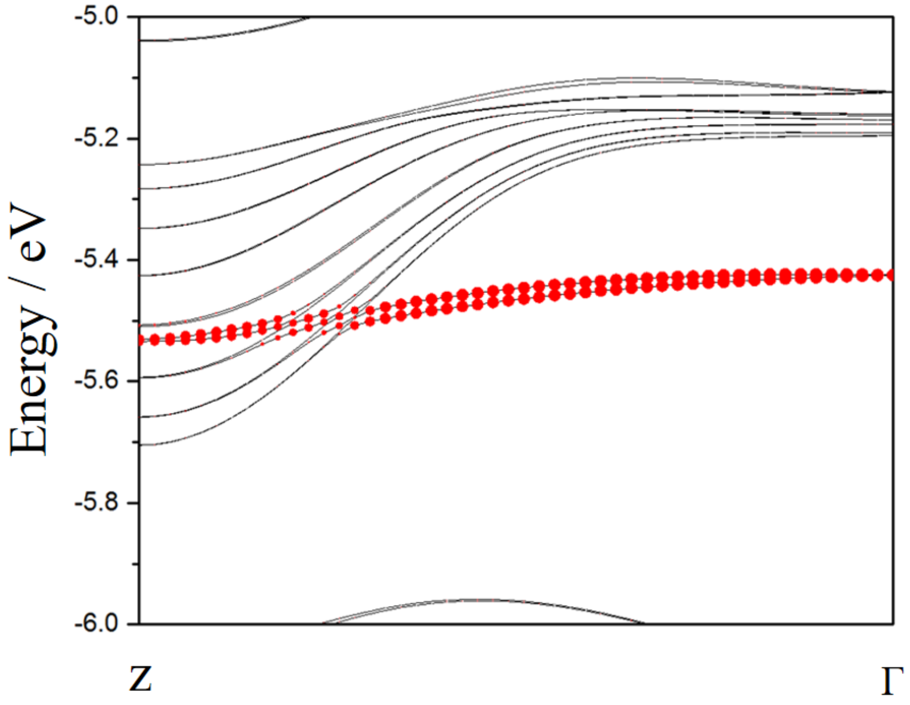

Figure S21. The H orbital after accept electrons from the surface states.

Catalyst	*j* / mA cm^{-2}	Overpotential mV	Tafel slope mV/dec.	References
$PtSn_4$ single crystal	10	37	39	This work
np-CuTi	10	47	110	Nat. Comm. 6, 6567 (2015)
Au-Ru nanowire	10	50	30.8	Nat. Chem. 2018
$Co(OH)_2/Pt$	10	248	N/A	Nat. Mater. 11, 550-557 (2012).
CoN_x/C	10	170	75	Nat. Commun. 6, 7992 (2014).
MoC_x nano-octahedrons	10	142	53	Nat. Comm. 6, 6512 (2015)
$NiFeO_x$ nanoparticles	10	88	150	Nat. Commun. 6, 7261 (2015)
CoP nanowire	10	209	129	J. Am. Chem. Soc 136, 7587-7590 (2014)
Mo_2C nanotubes	10	112	55	Angew. Chem. Int. Ed. 54, 15395-15399 (2015)
CoPS nanoplate	10	48	57	Nat. Mater. 14, 1245–1251 (2015)
$Ru/C_3N_4/C$	10	79	N/A	J. Am. Chem. Soc. 138, 16174-16181 (2016)
$NiCo_2O_4$ hollow microcuboids	10	110	49.7	Angew. Chem. Int. Ed. 55, 6290-6294 (2016).
Ni-Mo nanopowders	20	70	N/A	ACS Catal. 3, 166-169 (2013)
MoP nanoparticles	10	130	48	Energy Environ. Sci., 7, 2624–2629(2014)
VOOH hollow nanospheres	10	164	104	Angew. Chem. Int. Ed. 129, 588-592 (2017)
Pt_3Ni frame/$Ni(OH)_2/C$	12.6	100	N/A	Science 343, 1339-1343 (2014)

Table S5. Summary of some recently reported representative HER electrocatalysts in basic electrolytes.

References

[1] E. Mun, H. Ko, G. J. Miller, G. D. Samolyuk, S. L. Bud'ko, P. C. Canfield, *Phys. Rev. B* **2012**, *85*.

[2] aJ. Kibsgaard, T. F. Jaramillo, *Angewandte Chemie* **2014**, *53*, 14433-14437; bR. Zhang, X. Wang, S. Yu, T. Wen, X. Zhu, F. Yang, X. Sun, X. Wang, W. Hu, *Adv Mater* **2017**, *29*; cY. Zheng, Y. Jiao, Y. Zhu, L. H. Li, Y. Han, Y. Chen, M. Jaroniec, S. Z. Qiao, *J Am Chem Soc* **2016**, *138*, 16174-16181.

[3] B. Hinnemann, P. G. Moses, J. Bonde, K. P. Jørgensen, J. H. Nielsen, S. Horch, I. Chorkendorff, J. K. Nørskov, *J. AM. CHEM. SOC.* **2005**, *127*, 5308-5309.

[4] E. Skúlason, V. Tripkovic, M. E. Björketun, S. Gudmundsdóttir, G. Karlberg, J. Rossmeisl, T. Bligaard, H. Jónsson, J. K. Nørskov, *J Phys. Chem. C* **2010**, *114*, 18182-18197.

[5] P. W. Atkins, **1998**, pp 1485, 1925, and 1942.

Author Contributions

G. L. and C.F. conceived and designed the experiments under the supervision of Cl. F., W. S., Q. Y., and Y. S. contributed the theoretical investigation. L. J. performed the STM measurement. J. W., X. L, and M. F. conducted the XPS measurement. R. S., A. S., and S. P. carried out the TEM measurement. M. K. and G. B. contributed to the single crystal XRD. A. Y., N. K., J. Z., X. F., J. G., G.A., and V. M. assisted with the data analysis. G. L. wrote the paper. All authors discussed the results and commented on the manuscript.
